# A rare case of azygous vein aneurysm with cotriatrium dextrum

**DOI:** 10.1093/jscr/rjac413

**Published:** 2022-09-19

**Authors:** Ashviini Chandrakumanan, Sui Wu Tee, Melvin Sylverster, Avisha Richards, Loon Guan Chew

**Affiliations:** Department of General and Vascular Surgery, Serdang Hospital, Selangor, Malaysia; Department of General and Vascular Surgery, Serdang Hospital, Selangor, Malaysia; Department of General and Vascular Surgery, Serdang Hospital, Selangor, Malaysia; Department of General and Vascular Surgery, Serdang Hospital, Selangor, Malaysia; Department of General and Vascular Surgery, Serdang Hospital, Selangor, Malaysia

## Abstract

Azygous vein aneurysm (AVA) is an infrequent entity for posterior mediastinal lesion and paratracheal mass. Usually asymptomatic, AVA is discovered during routine examination of a patient. The patho-etiology of the azygous vein aneurysm has not been fully understood till date, making it difficult to postulate the most common cause for its occurrence. Nonetheless, AVA has to be taken into consideration as a differential diagnosis for posterior mediastinal mass or right paratracheal lesion. The objective of this paper is to report a rare case of AVA and further discuss on its patho-etiology leading to the dilatation of azygous vein aneurysm.

## INTRODUCTION

Azygous vein aneurysm (AVA) is an infrequent entity for posterior mediastinal lesion and paratracheal mass. Usually asymptomatic, AVA is discovered during routine examination of a patient. The patho-etiology of the azygous vein aneurysm has not been fully understood till date, making it difficult to postulate the most common cause for its occurrence. Nonetheless, AVA has to be taken into consideration as a differential diagnosis for posterior mediastinal mass or right paratracheal lesion. The objective of this paper is to report a rare case of AVA and further discuss on its patho-etiology leading to the dilatation of azygous vein aneurysm.

## CASE REPORT

A middle-aged gentleman was referred for an incidental finding of AVA. He initially presented with intermittent chest discomfort post strenuous activity. However, he denied of any shortness of breath, palpitation, postural hypotension, cough, dysphagia or hoarseness of voice. He had no other significant medical co-morbidities, and no recent trauma. He was asymptomatic during review; physical examination and laboratory tests were unremarkable. Investigations performed including electrocardiogram and blood investigations were unremarkable. An echocardiography was then performed and cotriatrium dextrum was identified.

**Figure 1:**
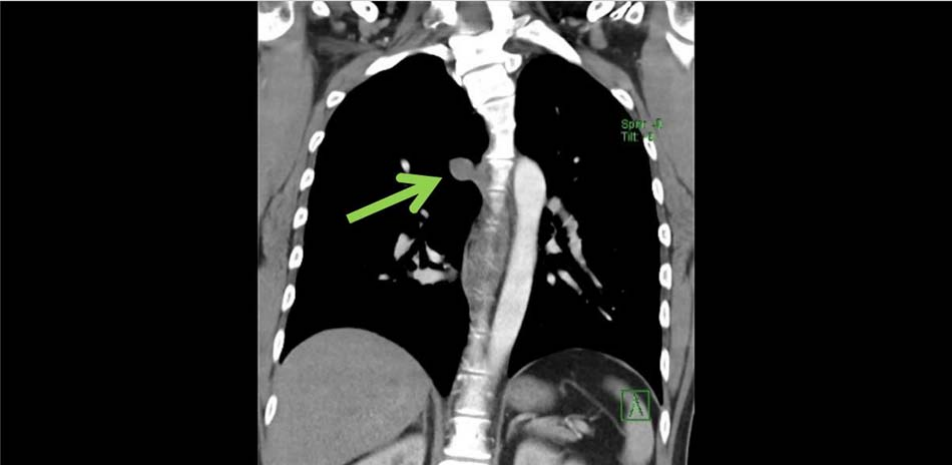
Coronal view of azygous vein aneurysm.

**Figure 2:**
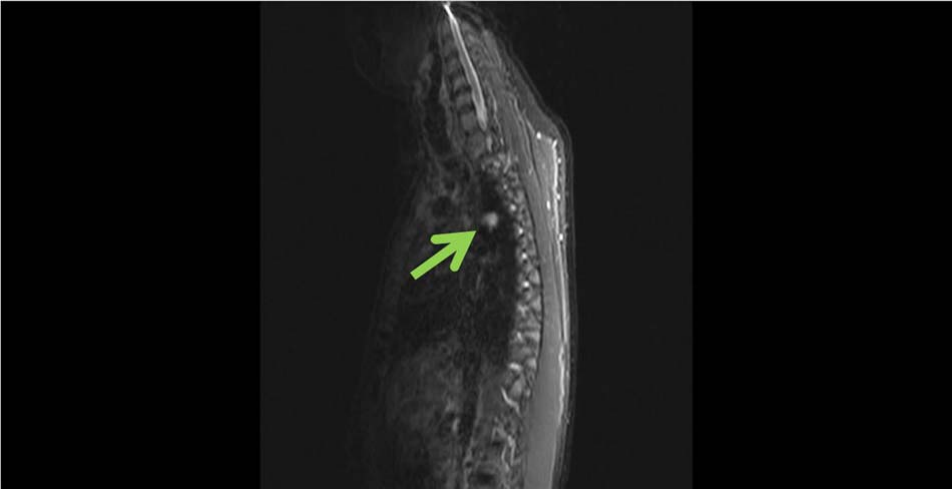
Sagittal view of azygous vein aneurysm.

Subsequently cardiac magnetic resonance imaging (MRI) established the presence of a well-defined lobulated hyperintense HASTE lesion at the right paraspinal lesion, posterior to the carina at T4/T5 level, measuring 1.2 cm × 3.1 cm × 1.5 cm. (Figs 1 and 2) There was no regional wall motion abnormality, ventricular strain, valvular incompetency or pericardial effusion. Further contrast enhanced computed tomography (CECT) thorax with complementary MRI thoracic spine confirmed the location of the lesion, and it appeared to be in continuity with the adjacent AV. On the complementary MRI, the lesion showed isointense signal on T1, hyperintense signal on T2 and T1RM. On CINE sequence, there was continuous blood flow within this lesion to the AV. A diagnosis of AVA was made.

Further abdominal ultrasonography revealed a normal liver, no varices or signs of portal hypertension. As no definite cause was identified and it was a relatively small aneurysm, conservative management was decided following multidisciplinary team discussion. An oesophagogastroduodenoscopy (OGD) was recommended to ensure rule out the pathology of AVA.

## DISCUSSION

An aneurysm has been defined as localized abnormal dilatation of blood vessels, 1.5 times larger in diameter than that of adjacent vessel according to guideline of joint vascular societies. Although aneurysm of an artery is common, AVA is rare.

The AV is a cranial continuation that is formed from the union of right subcostal and right ascending lumbar veins in the thorax. Known as a unilateral vessel that has valves, AV carries deoxygenated blood to the superior vena cava (SVC), arches at the level of T4, anterior to the vertebral column and subsequently empties into the SVC. The normal diameter of AV is delineated as no >1 cm, nevertheless the arch may dilate up to 15 mm in pregnancy as a result of hypervolemia [1].

There are three pairs of veins that evolve to form the azygous system, namely precardinal, postcardinal and supracardinal. The azygous system develops from the paired supracardinal veins, where the proximal segment of right supracardinal vein joins with the posterior cardinal veins to form the arch of azygous system. The left supracardinal vein becomes the hemiazygous and accessory hemiazygous vein.

Because of its rarity, the etiology of AVA has not been fully understood till date. Kreibich *et al.* categorized AVA into idiopathic, acquired due to pressure or volume overload and traumatic AVA [2]. Idiopathic AVA is generally considered congenital in origin, most commonly affecting the arch of the AV as it is the weakest anatomical point for the formation of aneurysm [2].

Infrahepatic interruption of inferior vena cava (IVC) with azygous and hemiazygous is infrequent and commonly occurs in association with congenital cardiac defect. It occurs in 0.3% of healthy and 0.6% of patients with cardiac disorder such as situs inversus, polypenia syndrome, deep vein thrombosis and sick sinus syndrome [4]. Interruption of IVC below the hepatic occurs when there is a failure of the right subcardinal-hepatic anastomosis, along with atrophy of right subcardinal vein. Hence, systemic flow beyond this point is accommodated by dilated AV, the IVC drains into the arch of AV, which subsequently drains into SVC at the usual anatomical location-right paratracheal area, and the hepatic veins drains independently through diaphragm into the right atrium [3, 4].

Superior vena cava obstruction may also result in AV dilatation. About 90% of the SVC obstructions are secondary to malignancy, most commonly right sided carcinoma occurring in the thoracic cavity such as lymphoma [5]. This results in collateral pathway that drains via the azygous in high flow system leading to its dilatation. Depending on the level of obstruction, the flow to the AV can be antegrade or retrograde.

Another common entity of pathology is portal hypertension. Portal pressure is the product of intrahepatic vascular resistance along with the portal blood flow. An increase in portal pressure (>10 mmHg) results in the dilatation of the left gastric vein, because of retrograde blood flow and subsequent dilatation of the venous esophageal tributaries of the left gastric vein, which eventually leads to the dilatation of the AV [6].

Various cardiac causes have also been attributed to the dilatation of AV. Right ventricular strain, tricuspid insufficiency, constrictive pericarditis, severe right tricuspid valve regurgitation and pericardial effusion leads to right heart failure, increasing the right atrial and central venous pressure eventually increasing blood flow to AV.

## CONCLUSION

In this case, we believe the finding of cotriatrium is the leading factor for the dilatation of AV. During fetal life, the right sinus venous would regress to form crista terminalis, Thebesian valve and Eustachian valve. Failure of regression of sinus venosus leads to partitioning of the atrium into two chambers, leading to cranial vena cava obstruction. This increases the pressure and leads to the shunting of blood via the AV [7]. The potential explanation of his chest discomfort may be from the increased pressure flow within the cranial vena cava from his strenuous physical activity, leading to increased blood flow, right to left shunt through the aberrant AV.
